# Reproductive Toxicity: Too Much of a Good Thing?

**Published:** 2006-10

**Authors:** Valerie J. Brown

Pregnant women are famously exhorted to faithfully take their daily prenatal vitamins, which often contain iron and other minerals. But new research suggests that a weekly iron supplement coinciding with the renewal of the small intestine’s mucosal lining cells (where nutrient absorption occurs) works better than a daily supplement and prevents problems resulting from too much iron at the wrong times.

Maternal iron deficiency and anemia early in gestation can result in premature birth and low birth weight. These, in turn, can trigger further problems ranging from slow physical growth and motor development to impaired emotional control. In severe cases, both maternal and fetal survival can be threatened at or near birth. Thus, there exists a near-global public health policy of maternal iron supplementation during pregnancy.

The new study appears in the July 2006 issue of *Archives of Medical Research*. A team including nutritionist and epidemiologist Esther Casanueva of the National Institute of Perinatology Isidro Espinosa de los Reyes (INPerIER) in Mexico City and colleagues elsewhere in Mexico City and California studied 116 women receiving prenatal care at INPerIER. All had come to INPerIER for prenatal care by gestational week 20.

None of the women were anemic at that point, but 66% had low levels of ferritin (the principal form of stored iron), suggesting low iron nutritional status. Half took 60 mg of iron as ferrous sulfate with 200 μg of folic acid and 1 μg of vitamin B_12_ once a day; the others took double this dose once a week. The researchers checked the women’s levels of hemoglobin (which transports oxygen) and ferritin every four weeks through the end of pregnancy.

More of the women taking the weekly dose were mildly anemic (with hemoglobin levels shown not to carry any risk for mothers and infants) compared with the women taking the daily dose. However, by weeks 28 to 36, women taking the daily supplement had a significantly higher prevalence of hemoconcentration, a condition defined as hemoglobin levels above 145 g/L. Ironically, both early gestational iron-deficiency anemia and hemoconcentration later in pregnancy increase the risk of premature birth and low birth weight. Thus, the researchers suggest that excess iron supplementation can cause the same problems it is supposed to correct.

Animal studies suggest that excess iron can also trigger formation of free radicals in the intestinal mucosa and other tissues, and that both iron deficiency and iron overload can damage nuclear DNA and mitochondrial DNA. This kind of damage has been implicated in cancer induction.

The intestinal mucosa is renewed every 5 to 6 days and will absorb as much iron as necessary to maintain iron balance; however, mature cells will stop absorbing iron entirely if they are flooded with it, even if there is an iron deficit. “Maintaining a high iron environment in the intestine by ingesting significantly more iron than needed every day overwhelms this safety system,” says coauthor Fernando E. Viteri. A more subtly calibrated iron supplementation during pregnancy may be as effective as current public health recommendations, and perhaps safer.

